# Uncovering the Invisible: The Role of High-density Catheters in Recognizing Fractionated Signals in Pulmonary Vein Isolation

**DOI:** 10.19102/icrm.2024.15063

**Published:** 2024-06-15

**Authors:** Harini Lakshman, Ammar Ahmed, Steven Coutteau, Dipak Shah

**Affiliations:** 1 Department of Cardiovascular Medicine, Ascension Providence Hospital, Southfield, MI, USA; 2 Abbott, Green Oaks, IL, USA; 3 Department of Electrophysiology, Ascension Providence Hospital, Southfield, MI, USA

**Keywords:** Atrial fibrillation, high-density grid catheter, mapping

## Abstract

The HD Grid multipolar mapping catheter has emerged as an invaluable tool for greater effectiveness of pulmonary vein isolation (PVI). In the cases described here, fractionated signals seen with the HD Grid catheter at the left atrial appendage (LAA) and left superior pulmonary vein (LSPV) junction were ablated. These signals are not likely to be visualized with conventional catheters and may cause recurrences due to incomplete PVI. The directional sensitivity limitations of bipolar electrogram recordings and the unique anatomy of the LAA–LSPV ridge further contribute to the challenge of evaluating PVI. The HD Grid catheter’s ability to record bipoles parallel and perpendicular to the catheter splines and its high-density mapping capabilities provide a superior means for identifying gaps in ablation and detecting the low-voltage isthmus. Furthermore, factors such as ablation quality, catheter stability, and thickness of the LAA–LSPV ridge influence the presence of fractionated signals and the success of PVI. Incorporating preprocedural imaging modalities, such as computed tomography or magnetic resonance imaging, and real-time intracardiac echocardiography could enhance the tailored approach to address these challenges. Future developments in the HD Grid technology, including the option for contact force measurement during mapping, may offer additional insights into the nature of these signals. This case series highlights the significance of using the HD Grid catheter for a detailed interrogation of the LAA–LSPV ridge, ultimately leading to more effective PVI and improved outcomes in patients with atrial fibrillation.

## Introduction

Atrial fibrillation (AF) ablation has significant recurrence rates, which vary from 35%–60% in the literature.^1^ In 98% of patients with recurrence, pulmonary vein (PV) reconnection is the likely mechanism.^2^ The entrance block is the most common endpoint signifying PV isolation (PVI), and circular mapping catheters are often used to confirm this. With the advent of higher-fidelity catheters, such as the high-density grid (HD Grid) catheter, the entrance block previously seen with circular catheters is often not present.^3^

We present a case series of fractionated signals at the ridge between the left superior pulmonary vein (LSPV) and the left atrial appendage (LAA), which were detected by the HD Grid catheter, which would likely be overlooked by circular mapping catheters, leading to recurrence of AF.

## Case presentations

**Figure 1:**
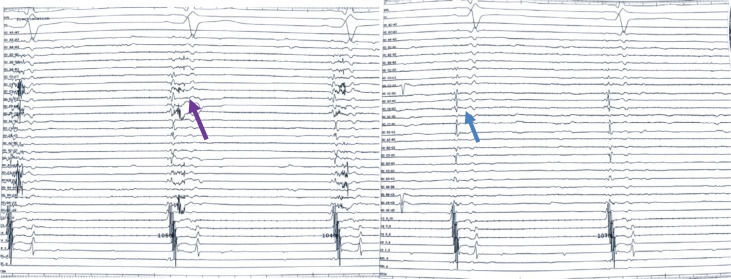
Case 1: Presence of fractionated signals between the left superior pulmonary vein and left atrial appendage as seen with HD Grid catheter mapping after atrial fibrillation ablation (indicated by a purple arrow) and post-ablation electrogram with no residual signals, as indicated by a blue arrow.

**Figure 2:**
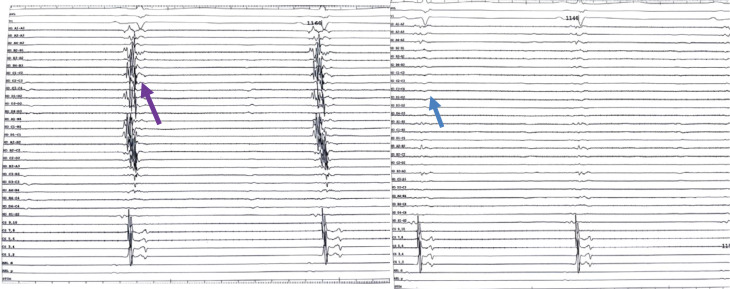
Case 2: Fractionated signals identified by the HD Grid as indicated by a purple arrow; post-ablation, the absence of fractionated signals is indicated by a blue arrow.

**Figure 3:**
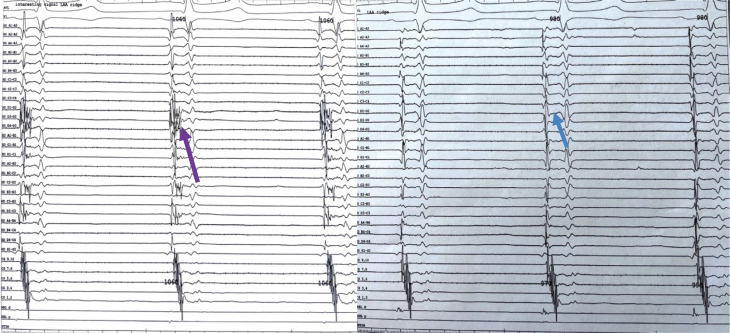
Case 3: Fractionated signals identified by the HD Grid as indicated by a purple arrow; post-ablation, the absence of fractionated signals is indicated by a blue arrow.

As seen in **Figures 1–3**, each of the included cases underwent paroxysmal AF ablation with PVI after ensuring contact forces of >8 *g*. The baseline characteristics are provided in **Table 1**.

After ensuring that all the PVs were isolated, by demonstrating an entrance block, the Advisor™ HD Grid Mapping Catheter, Sensor Enabled™ (Abbott, Chicago, IL, USA) was used to map and confirm the exit block. There was the presence of fractionated signals between the LSPV and LAA in each of these cases, which would not be uncovered if using a conventional circular mapping catheter.

Ethical approval was not required given the retrospective nature of the essential procedures.

## Discussion

In the series of cases described **(Figures 1–3)**, fractionated signals seen with the HD Grid catheter at the LAA and LSPV junction were ablated. These signals would not likely have been visualized with conventional catheters and may cause recurrences due to incomplete PVI.

The directional sensitivity of bipolar electrogram recordings is a limitation. When the wavefront is propagating parallel to the electrode pair, higher amplitudes are recorded than when it moves perpendicularly.^1^ Considering that previous research demonstrated that myocardial fibers infiltrate the PVs longitudinally, circumferentially, and obliquely, this limitation of typical bipoles may be crucial for evaluating PVI.^4^

**Figure 4:**
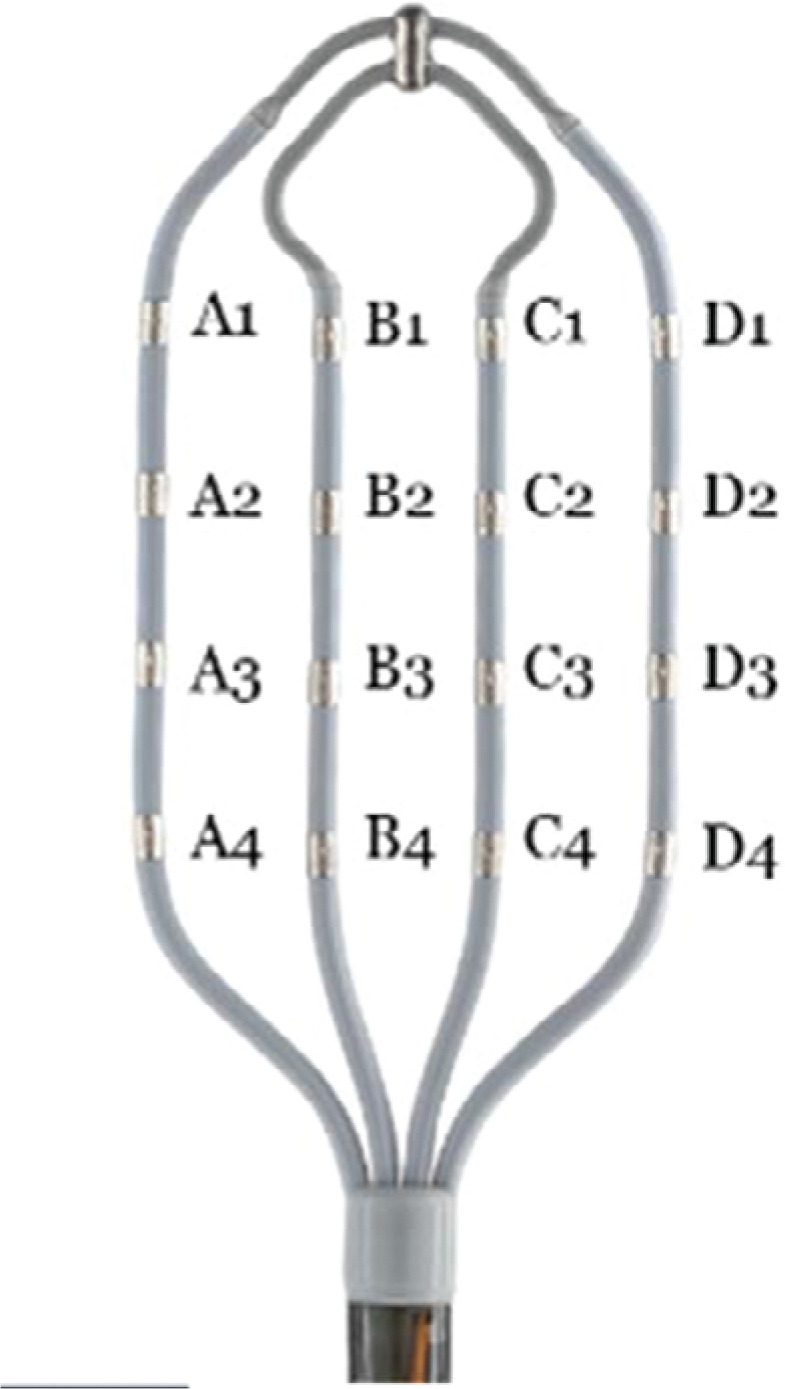
Abbott’s Advisor™ HD Grid Mapping Catheter, Sensor Enabled™ product with the electrodes labeled.

In contrast to conventional multipolar mapping catheters and ablation catheters, which only permit bipole recording parallel to the splines, the HD Grid multipolar mapping catheter is a novel and one-of-a-kind multipolar mapping catheter that permits bipole recording parallel and perpendicular to the splines via 16 electrodes (**Figure 4**).^4^

Studies comparing the HD Grid catheter with circular mapping catheters, cryoablation catheters, and pacing the ablation line found the HD Grid catheter to be superior in identifying the gaps in ablation.^4–7^ Approximately 52.5% of patients with recurrent AF had gaps in ablation detected by HD Grid compared to traditional catheters. The HD Grid offers the capability to perform high-density mapping to identify the anatomical substrate and low-voltage isthmus, which previously would have been missed.^8^

Other factors that could have resulted in these fractionated signals include the quality of ablation.

In our case series, at these locations, we had contact forces of >8 *g* and ablated with high-power, short-duration radiofrequency ablation (RFA) of 45 W for 10–25 s, targeting a lesion size index (LSI) of 6. Despite these standard RFA parameters, these signals were still present.^9^

The potential reasons why these signals were realized in our cases is that the HD Grid allows for excellent anterior flexion around the LAA and LSPV interface, which maximizes tissue contact. If flexion is not applied along the ridge, these signals will not likely be visualized. Future developments in the HD Grid technology potentially involving a contact force measure during mapping may allow operators to appreciate this more clearly.

The PV and LA junction is a complex interface surrounded by a wide range of heterogeneous tissue types.^1^ The location of the LAA in relationship to the LSPV as well as the thickness of the LAA–LSPV ridge make this area of ablation unique for each patient. Often, catheter stability in this region limits how antrally one can deliver RF. If the ablation is too ostial, these antral signals will be missed.

No preprocedural imaging was done in our cases. Possibly, computed tomography or magnetic resonance imaging could quantify the thickness of this region, and then a more tailored approach could be taken. Also, intracardiac echocardiography could potentially be used in real time to delineate the LAA–LSPV ridge. Depending on the anatomic issues visualized, possible options include a longer duration of RFA targeting a higher force–time integral or LSI, allowing for a transmural lesion or RFA on both the PV and LAA side of the ridge as a sandwich technique.

Our case series demonstrates a detailed interrogation of the LAA–LSPV ridge with the HD Grid as a culprit for incomplete PVI.

## Figures and Tables

**Table 1:** Baseline Characteristics

Case	Age (Years)	Sex	LA Size (mm^2^/BSA)	EF	Outcomes in 3 Months
1	62	M	38	50%–55%	No recurrence
2	69	M	41	55%–60%	No recurrence
3	71	F	40	55%–60%	No recurrence

*Abbreviations:* BSA, body surface area; EF, ejection fraction; LA, left atrial.
